# Overview: Stress and Alcohol Use Disorders Revisited

**DOI:** 10.35946/arcr.v34.4.02

**Published:** 2012

**Authors:** Robert M. Anthenelli

**Affiliations:** **Robert M. Anthenelli, M.D.,***is associate chief of staff for Mental Health, Veterans Affairs, San Diego Healthcare System; professor and vice chair for Veterans Affairs; and director, Pacific Treatment and Research Center, Department of Psychiatry, School of Medicine, University of California, San Diego, California.*

**Keywords:** Alcohol consumption, alcohol use disorders, stress as a cause of alcohol and other drug use, stress, stressors, stress response, stress reactivity, physiological response to stress, brain, genetic factors, environmental factors, allostasis, allostatic load, allostatic state, homeostasis

## Abstract

Nearly 13 years have passed since *Alcohol Research & Health* (now titled *Alcohol Research: Current Reviews*) first visited the topic of “Alcohol and Stress.” Since that time, the field has advanced considerably. New terms have been developed to describe the complex physiological interactions that occur when an individual is faced with stressful events and more is known about how the brain and body work to offset the changes induced through stress-response mechanisms. An individual’s reactions to stress vary according to a number of factors, such as his or her genetic makeup, environment, life events, gender, age, and type and duration of stress. Drinking alcohol has the unique ability to both relieve stress and to be the cause of it, creating in a sense a double-edged sword. Understanding the link between alcohol drinking, stress, and alcohol use disorders (AUDs) is a critical area for ongoing investigation. Discoveries emanating from this field not only add to the burgeoning literature on stress and the risk for disease but also may provide answers to help prevent and intervene in the development of AUDs.

In the 13 years since *Alcohol Research & Health* (now titled *Alcohol Research: Current Reviews*) first visited the topic of “Alcohol and Stress” (see Vol. 23, No. 4, 1999), there has been a sustained flow of new information in the field prompting us to publish this updated edition. Indeed, one could argue that this second look at the topic is long overdue. An entirely new lexicon of terms^1^ has been developed to capture our evolving conceptualization of stress and its effects on health and disease risk. Many of these terms (e.g., allostasis and allostatic load), which were becoming popular around the turn of the 21st century, were hardly mentioned in that previous edition, so there is a fair amount of catching up to do. Unthinkable events (e.g., the 9/11 terrorist attack and its aftermath—Operation Enduring Freedom and Operation Iraqi Freedom) have occurred, spurring renewed interest in the role of uncontrollable acute and chronic stressors on drinking behaviors in civilians and military personnel alike. New fields have emerged (e.g., epigenetics), and their findings demonstrate that early-life trauma can leave an indelible stamp on an individual’s genetic makeup (i.e., genome) and stress circuitry. Gene–environment interactions have been discovered that partly quell the artificial argument as to whether nature or nurture most influences disease risk. Finally, new integrated treatments have emerged (e.g., Najavits’ Seeking Safety), and mechanisms of action partly defined (e.g., naltrexone’s effects on stress axis function), that demonstrate how understanding the links between stress and alcohol drinking promotes improved treatment options for patients with alcohol use disorders (AUDs).

In their opening article to the 1999 *Alcohol Health & Research* edition on “Alcohol and Stress,” Anisman and Merali (1999) summarized the literature to develop a working definition of stress and stressors (i.e., stressful situations) that we attempt to update in the present treatise. We also will embellish upon several themes that these authors chose to highlight, including the importance of sex differences and stressor specificity. By introducing these themes, we hope to set the stage for the articles that follow, which delve into several of these topics more deeply.

## What Is Stress?

Webster’s *Third New International Dictionary* (1981, p. 2260) defined stress as “a physical, chemical, or emotional factor (as trauma, histamine, or fear) to which an individual fails to make a satisfactory adaptation, and which causes physiologic tensions that may be a contributing cause of disease.” Although this term now is widely used in the common vernacular, it is interesting to note that the scientific conceptualization of this phenomenon dates back only about 150 years.

Most stress research historians agree that the French physiologist, Claude Bernard (1865), was the first to recognize a key element in the stress response—the phenomenon now known as feedback regulation. Bernard noticed that the internal environment of cells (“milieu intérieur”) is tightly regulated and largely dependent on feedback it receives from the periphery or “external environment” (Goldstein and Kopin 2007). Some 65 years later, Sir Walter Cannon coined the term “homeostasis” to capture the “coordinated physiological processes that maintain most of the steady states of the organism” (Cannon 1929 as cited by Goldstein and McEwen 2002, p. 55). From Cannon’s perspective, which derived from his study of the sympathetic nervous system (he also coined the phrase “fight-or-flight responses”), all organisms adjusted to challenges to their internal environments by making compensatory responses intended to restore homeostasis. By accomplishing such, the organism’s chances for survival improved because the homeostatic or steady state was viewed as optimal and fixed at some preordained, stable level (Goldstein and Kopin 2007; Neylan 1998).

The Hungarian scientist, Hans Selye, who was influenced by Cannon’s work, developed the concept of the General Adaptation Syndrome in 1936. Selye’s theories, which dominated thinking on the nature of the stress response for more than 50 years, hypothesized that a classical syndrome developed in all organisms “the symptoms of which are independent of the damaging agent or the pharmacological type of the drug employed” (Selye 1936, p. 32). He further hypothesized that this stress response had three stages: an initial alarm reaction (akin to Cannon’s fight-or-flight response) that involved the release of anterior pituitary hormones; a second, adaptation phase, wherein an attempt is made to resist the stressor; and a third, exhaustion phase, which, at its extreme, could lead to death of the organism (Goldstein and Kopin 2007; Selye 1936).

Over time, scientists began challenging two key concepts in this definition of stress as any real or imagined threat to homeostasis (McEwen and Stellar 1993). First, Selye’s assertion that stress responses were uniform and generalized regardless of stressor type was modified in recognition that certain types of stressors (e.g., physical versus emotional, see below) evoked activation of specific effector systems. For example, exposure to extreme cold produces a marked activation of the sympathetic noradrenergic system in an effort to regulate core body temperature, yet it has minimal effects on the endocrine or hormonal stress response (Goldstein and Kopin 2007). Thus, Selye’s doctrine of a unitary, nonspecific stress response gave way to a more refined view that individuals activate stress systems more selectively depending on the characteristics of the stressor.

Second, scientists began recognizing that physiological regulatory systems spanned multiple domains, were dynamic and not static, and fluctuated constantly based on the animal’s biological rhythms and physiological demands. Moreover, the notion that there existed some static, ideal, homeostatic set point gave way to thinking that, instead, these set points vary across a dynamic operating range which change over time. Thus, Sterling and Eyer ([1988] as cited in McEwen and Stellar 1993) coined the term allostasis to describe this operating range and the organism’s ability to increase or decrease body functions to a new steady state when challenged.

McEwen and Stellar (1993) embellished on the principle of allostasis by defining a new concept that these authors labeled allostatic load. This term connotes the toll placed on individuals when they have to constantly or repeatedly adjust the operating range to maintain fluctuating set points. This “wear and tear” can predispose the individual to disease, especially in the context of chronic stress.

It is interesting to note that in this seminal paper, the authors cite “the reciprocal relationship between stress and alcohol consumption” as an example of allostatic load:

In short, whereas drinking may help the person cope with stress in the short-term, there is a longer-term cost. As the person tries to balance the reciprocal effects of stress and alcohol consumption in this manner, the upward spiral of both stress and drinking increases this overall cost (allostatic load) both behaviorally and biologically (McEwen and Stellar 1993, p. 2096).

In summary, although the use of the term stress has become commonplace, the scientific conceptualization of this state is a relatively recent phenomenon and is still evolving. Stress has been broadly defined “as a threat, real or implied, to the psychological or physical integrity of an individual” (McEwen 2000, p. 108). Other terms, however, such as allostasis (“maintaining stability, or homeostasis, through change” [Sterling and Eyer 1988 as cited in McEwen 2000, p. 108]) and allostatic load (“the price the body pays for being forced to adapt to adverse psychosocial or physical situations” [McEwen 2000, p. 110]) are newly emerged and are helping to better define the relationships between stress and disease risk, including the risk for AUDs, as described below.

## Stress and Addiction to Alcohol and Other Drugs

Koob and Le Moal (1997, 2001) began formally linking the brain’s stress and reward systems in an allostatic model of alcohol and other drug addiction that still holds sway over the field today. As described in detail elsewhere (Koob and Le Moal 1997, 2001) and alluded to in Koob and colleagues’ contribution in this edition (see pp. 516–521), these scientists hypothesized that alcohol and other drug addiction represents an allostatic state whereby an individual’s hedonic set point has drifted downward and been recalibrated at a new point below the normal, homeostatic range. The fluctuating hysteresis of this proposed downward sloping “mood” curve reflects the operating range of the brain’s reward and stress systems, which engage in a struggle to adjust and readjust in the setting of repetitive alcohol (or other drug) use. Thus, in this allostatic model, alcohol drinking can be viewed as both a reward and a stressor—an interpretation which is consistent with observations that acute doses of alcohol simultaneously increase brain concentrations of mesolimbic dopamine and other reinforcing neurotransmitters as well as brain levels of corticotropin-releasing factor (CRF) and blood levels of adrenocorticotropin hormone (ACTH) and cortisol, the major stress hormones in the brain and body (Rivier and Lee 1996).

At first glance, this notion of alcohol and other drugs of abuse working as stressors (i.e., taxing to the individual) flies in the face of the more commonly held belief that ethanol has stress-response–dampening effects. However, several characteristics of the drug may explain this paradox. First, alcohol’s rewarding properties may counterbalance or mask its stress-provoking effects. This happens on a number of different levels: (1) the drug produces brain depressant effects by acutely enhancing GABAergic tone, while inhibiting excitatory glutamatergic signaling; (2) ethanol acutely enhances the release of reinforcing neurotransmitters (e.g., dopamine and endogenous opiates) and neuromodulators (e.g., endocannabinoids); and (3) alcohol’s effects on the release of the stress hormone, cortisol, in the periphery triggers further rewarding properties in the brain (see the article by Stephens and Wand, pp. 468–483).

Second, consistent with the second phase in Selye’s general adaptation syndrome, and the opponent-process model (Solomon and Corbit 1973) evoked in Koob and Le Moal’s allostatic model of addiction, the brain resists or adapts to repeated, alcohol-induced stress hormone elevations. This neuroadaptation underlies the allostatic change associated with chronic heavy drinking and manifests as a blunted stress response in recently abstinent alcoholics (see Stephens and Wand, pp. 468–483).

In summary, although low doses of alcohol in non–alcohol-dependent individuals produce rewarding effects that are perceived to attenuate stress, in actuality, the drug stimulates the release of CRF and stress hormones. Chronic, heavy use of ethanol produces an allostatic state wherein reinforcing and stress-provoking effects of the drug battle and oppose each other but generally contribute to an altered set point below that associated with normal mood states. When repeated over many months to years, this struggle exerts its toll (i.e., produces allostatic load) on the brain and body, as there is a cost associated with the chronic efforts to adapt to these stressors. Thus, drinking to relieve stress proves to be a double-edged sword.

## Factors Influencing Stress Reactivity

Casual readers of the alcohol and stress literature can become frustrated by the apparent lack of uniformity of findings. For example, when analyzing studies attempting to determine whether stress leads to relapse to alcoholism (see the article by Thomas and colleagues in this edition, pp. 459–467), readers will observe that sometimes blunted hormonal responses are associated with increased relapse risk; whereas, in other instances, exaggerated hormonal responses predict the return to drinking. However, when one considers that stress responsivity is governed by a host of factors related to (1) the characteristics of the stressor and (2) the characteristics of the individual, some of this heterogeneity in findings can be explained.

### Stressor–Specificity

Painstakingly detailed neuroanatomical studies in experimental animals were among the first to demonstrate that organisms have evolved different stress circuits to adapt to life’s variety of stressors (Goldstein and Kopin 2007; Pacak et al. 1998). This stressor-specific strategy certainly makes sense from an evolutionary standpoint: it would be extremely inefficient to mobilize the same effector systems to keep an animal’s core temperature up when exposed to cold weather as it would to respond to hemorrhagic hypotension. However, there also is an advantage to having some redundancy across these effector systems. For example, the hypothalamic–pituitary–adrenal (HPA) axis, which mediates the endocrine or hormonal response to certain stressors, interconnects with the adrenomedullary hormonal system and the sympathetic noradrenergic system (SNS). This does not mean, however, that specific stressors activate all three effector systems to the same extent. Thus, when researchers measure the outputs of these effector systems (i.e., ACTH and cortisol in the bloodstream of humans to monitor HPA axis reactivity versus heart rate and blood pressure which reflect SNS activity) in response to various stress paradigms they may not necessarily find unanimity of responses.

Scientists have conceptualized different categories of stressors to better capture this phenomenon. Thus, distinctions such as “psychogenic versus neurogenic,” “processive versus systemic” (see the article by Herman, pp. 441–447), and “physical versus psychological versus pharmacologic” stress have been used to describe the various stress induction paradigms used in experimental animals and humans (for a partial list, see table 1). The stress-response patterns generated by these different types of stressors are not uniform, however, which is a point frequently lost among casual observers.

## Other Stressor Characteristics

In addition to the types of stressors influencing stress reactivity, there are other features associated with the stressful experience that affect an individual’s responsiveness (see table 1). For example, the degree of controllability of the stressor influences response, with uncontrollable stress creating a greater level of response compared with events considered to be under an individual’s control (Anisman and Matheson 2005). It is interesting to note that even this seemingly behavioral, subjective phenomenon seems to be governed by stressor-specific neural circuits. For instance, experiments in rodents have demonstrated that the brain’s serotonin system seems to be of primary importance in modulating uncontrollable versus controllable stress (Hammack 2002). Whether a stressor is predictable or unpredictable influences the magnitude of the stress response, as does its duration (i.e., chronicity) (Anisman and Matheson 2005).

### Individual-Level Variables Affecting Stress Responsivity

Just as the type, predictability, and controllability of the stressor influence its response, an individual’s characteristics also affect stress reactivity. Of particular relevance to human-stress researchers is the individual’s gender, and a better understanding of this could help explain why women seem to develop AUDs following a stress-related condition, whereas the opposite temporal pattern applies for men (Kessler et al. 1997). Accumulating evidence indicates that women and men have evolved different stress-response activation patterns during the reproductive years (Kajantie and Phillips 2006) and that women respond more robustly to certain stressors than men and vice versa. For example, using one of the most popular psychological stress induction paradigms, the Trier Social Stress Test,^2^ several investigators have found that men react more robustly to this type of stressor than do women (Uhart et al. 2006). Additional evidence for this gender X stressor subtype interaction effect was found by Stroud et al. (2002), who reported that women mounted a greater stress response to a social evaluative stressor task (e.g., the participant feeling shunned by two confederate research associates feigning a spontaneous social interaction) than did men. Similarly, research has found gender- and stressor-specific effects to various pharmacological stress tests; women react more robustly to agents directly stimulating the pituitary gland or artificially lowering morning cortisol levels than do men, whereas men exhibit comparatively blunted responses to these manipulations (Anthenelli et al. 2009). Therefore, gender is an important variable to consider when evaluating how individuals react to certain stressors.

As described in other articles in this edition, an individual’s genetic makeup (see Schumann and colleagues, pp. 484–491), early-life experiences (see Brady and Back, pp. 408–413), environmental exposures to stress (see Keyes and colleagues, pp. 391–400), and predilection to anxiety and other psychiatric disorders (see Smith and Randall, pp. 414–431 and Schumm and Chard, pp. 401–407) can conspire to influence how adolescents and adults respond to stress and alcohol.

Heavy drinking and repeated withdrawal from alcohol may result in neuroendocrine changes that not only alter the body’s ability to respond to stressful challenges but also may undermine efforts to stop or reduce harmful drinking behavior (see articles by Alim and colleagues, pp. 506–515 and Becker, pp. 448–458).

Moreover, environmental insults can affect a person’s genetic architecture, and these epigenetic phenomena appear to influence the individual’s response to stressful life experiences and alcohol intake (see the article by Pandey and Moonat, pp. 459–467). When one considers that other personal characteristics such as an individual’s coping skills and social environment can modify how he or she reacts to stress, it should come as no surprise that laboratory paradigms in humans sometimes produce discrepant results (e.g., see Thomas and colleagues, pp. 459–467) in the literature.

## Conclusions

This brief overview sets the stage for the articles and sidebars that follow. In this issue, an esteemed group of alcohol and stress researchers tackle compelling questions such as “How Does Stress Lead to Risk of Alcohol Relapse?” (see the article by Sinha, pp. 432–440). Although the answers to important questions such as this are not fully known, what should shine through is how far the field has come since *Alcohol Research & Health* last tackled this topic. Understanding the relationships among alcohol drinking, stress, and alcohol use disorders is a critical area for ongoing investigation. Discoveries emanating from this field not only add to the burgeoning literature on stress and disease risk but also hold the promise to provide answers on how to prevent and intervene in this disorder. Here we offer a foundation for the next decade of discovery!

## Figures and Tables

**Table 1:** Factors Influencing the Stress Response

**Stressor type**
Processive (neurogenic or psychogenic)
Systemic (immune insults)
**Stressor characteristics**
Controllability
Predictability
Ambiguity/uncertainty
Chronicity
Intermittence
**Organismic variables**
Genetics
Age
Sex
**Experiential variables**
Previous stressor experiences (sensitization)
Early life events (maternal factors, trauma)
**Resource characteristics**
**Personal characteristics**
Coping skills
Self-esteem
Self-efficacy
Personality (hardiness, optimism, neuroticism)
And others
**Social characteristics**
Social support (perceptions)
Attachment (bonding)

SOURCE: Adapted from Anisman and Matheson 2005.

## Footnotes

1 For terms and their definitions, see the Glossary beginning on p. 522.

2 Using the Trier Social Stress Test, the subject is asked to give a speech and perform a simple math task in front of an audience. This test measures both social and cognitive stressors.
